# Comparison of accelerometer measured levels of physical activity and sedentary time between obese and non-obese children and adolescents: a systematic review

**DOI:** 10.1186/s12887-018-1031-0

**Published:** 2018-03-09

**Authors:** Rabha Elmesmari, Anne Martin, John J. Reilly, James Y. Paton

**Affiliations:** 1 School of Medicine, College of Medical, Veterinary, and Life Sciences, University of Glasgow, Glasgow, G12 8 QQ UK; 2 MRC/CSO Social and Public Health Sciences Unit, University of Glasgow, Glasgow, G12 8 QQ UK; 3 Physical Activity for Health Group, University of Strathclyde, George Street, Glasgow, G1 1XQ UK; 4 Al-Fatah Hospital, Medical School, Benghazi University, Benghazi, Libya; 5 Office Block, Ground Floor, Zone 1 (Paediatrics) Royal Hospital for Children, 1345 Govan Road, Glasgow, G51 4TF UK

## Abstract

**Background:**

Obesity has been hypothesized to be associated with reduced moderate-to-vigorous physical activity (MVPA) and increased sedentary time (ST). It is important to assess whether, and the extent to which, levels of MVPA and ST are suboptimal among children and adolescents with obesity. The primary objective of this study was to examine accelerometer-measured time spent in MVPA and ST of children and adolescents with obesity, compared with MVPA recommendations, and with non-obese peers.

**Methods:**

An extensive search was carried out in Medline, Cochrane library, EMBASE, SPORTDiscus, and CINAHL, from 2000 to 2015. Study selection and appraisal: studies with accelerometer-measured MVPA and/or ST (at least 3 days and 6 h/day) in free-living obese children and adolescents (0 to 19 years) were included. Study quality was assessed formally. Meta-analyses were planned for all outcomes but were precluded due to the high levels of heterogeneity across studies. Therefore, narrative syntheses were employed for all the outcomes.

**Results:**

Out of 1503 records, 26 studies were eligible (*n* = 14,739 participants; *n* = 3523 with obesity); 6/26 studies involved children aged 0 to 9 years and 18/26 involved adolescents aged 10.1 to19 years. In the participants with obesity, the time spent in MVPA was consistently below the recommended 60 min/day and ST was generally high regardless of the participant’s age and gender. Comparison with controls suggested that the time spent in MVPA was significantly lower in children and adolescents with obesity, though differences were relatively small. Levels of MVPA in the obese and non-obese were consistently below recommendations. There were no marked differences in ST between obese and non-obese peers.

**Conclusions:**

MVPA in children and adolescents with obesity tends to be well below international recommendations. Substantial effort is likely to be required to achieve the recommended levels of MVPA among obese individuals in obesity treatment interventions.

This systematic review has been registered on PROSPERO (International Database of Prospective Register Systematic Reviews; registration number CRD42015026882).

**Electronic supplementary material:**

The online version of this article (10.1186/s12887-018-1031-0) contains supplementary material, which is available to authorized users.

## Background

The prevalence of obesity among children and adolescents is now very high in both developing and developed countries [1, 2] and is a significant public health and clinical concern [3] that is attracting much research attention [4]. Obesity is known to have a significant impact on both physical and psychological health and children and adolescents with obesity face a number of health, social, and psychological problems [2, 5, 6]. Prevention of childhood obesity is a public health priority while treatment is becoming an increasingly important clinical issue.

A number of health behaviors have been associated with risk of obesity [7]. Poor diet, lack of physical activity (PA) and increased sedentary time (ST) have been linked to the development and maintenance of childhood and adolescent obesity [8–11]. Many evidence-based guidelines focusing on the amount of PA, particularly moderate–to-vigorous intensity physical activity (MVPA) required to produce health benefits, have been developed. These guidelines commonly recommend 60 min of MVPA as a daily minimum (7 days a week) for school-age children and adolescents [12–15].

Accelerometry currently represents the most accurate, inexpensive, and reliable method for objectively measuring both the amount and intensity of PA and amount of sedentary behavior (SB) [16, 17]. There have been many surveys and studies on the levels and adequacy of MVPA in healthy-weight children and adolescents [18, 19]. Since MVPA and ST are also important to health in those with obesity, and since obesity has been hypothesized to be associated with reduced MVPA [20] these variables need to be reviewed for children and adolescents with obesity. Whether and to what extent obesity in childhood and adolescence is associated with reduced objectively measured MVPA and ST/SB remains unclear, in part because of the lack of a synthesis of the evidence on this topic. Many studies have addressed the topic using subjective measurement methods, and/or considering the overweight as obese, and/or focusing on total volume of physical activity rather than MVPA. It is important to assess objectively measured time spent in MVPA and ST in children and adolescents with obesity. The primary aim of the present systematic review was therefore to determine obese children’s and adolescents’ habitual amount of time spent in MVPA, and examine whether those living with obesity met the current MVPA recommendation for health of a minimum of 60 min per day [14, 21]. Secondary aims were to examine time spent in accelerometer-measured SB by children and adolescents with obesity, and to determine whether MVPA and ST in obese children and adolescents were different from the non-obese peers.

## Methods

### Registration of systematic reviews

This systematic literature review was performed in accordance with the Preferred Reporting Items for Systematic Reviews (PRISMA) guidelines [22]. The review protocol was registered on PROSPERO (registration number CRD42015026882), the international prospective register for systematic reviews (http://www.crd.york.ac.uk/ NIHR_PROSPERO).

### Literature search

The literature search was conducted searching for English language peer-reviewed studies using the five most relevant electronic databases from 2000 up to March 2015 (accelerometry became more widely used in research from the early 2000’s): MEDLINE OVID; Cochrane library; EMBASE; SPORTSDiscus and CINAHL by AM. The literature search in the Cochrane Central Register of Controlled Trials is shown in Table 1, and was adapted as required for the other databases. Full literature search details are available from the corresponding author on request. The electronic search was complemented by reference citation tracking (forward and backward) of the included studies and of previous reviews.

**Table 1:** Search strategy of Cochrane Central Register of Controlled Trials

. #1 MeSH descriptor: [Child] explode all trees
. #2 MeSH descriptor: [Adolescent] explode all trees
. #3 child* or adolesc* or teen* or boy* or girl* or youth:ti,ab,kw (Word variations have been searched)
. #4 young near/1 (person or people):ti,ab,kw (Word variations have been searched)
. #5 #1or#2or#3or#4
. #6 MeSH descriptor: [Motor Activity] this term only
. #7 MeSH descriptor: [Exercise] explode all trees
. #8 MeSH descriptor: [Sports] explode all trees
. #9 MeSH descriptor: [Sedentary Lifestyle] explode all trees
. #10 physical* activ*:ti,ab,kw (Word variations have been searched)
. #11 exercis* or sport*:ti,ab,kw (Word variations have been searched)
. #12 active near/2 (living or lifestyle):ti,ab,kw (Word variations have been searched)
. #13 sedentary behavi?r:ti,ab,kw (Word variations have been searched)
. #14 (screen or sedentary or sitting or TV or television or computer or PC or video games) near/2 time:ti,ab,kw (Word variations have been searched)
. #15 #6or#7or#8or#9or#10or#11or#12or#13or#14
. #16 MeSH descriptor: [Accelerometry] explode all trees
. #17 acceleromet*:ti,ab,kw (Word variations have been searched)
. #18 actigraph*:ti,ab,kw (Word variations have been searched)
. #19 activity near/1 monitor*.:ti,ab,kw (Word variations have been searched)
. #20 #16or#17or#18or#19
. #21 MeSH descriptor: [Overweight] explode all trees
. #22 overweight or obes*:ti,ab,kw (Word variations have been searched)
. #23 #21or#22
. #24 #5 and #15 and #20 and #23

### Inclusion criteria

To be eligible for inclusion in the review, papers had to meet all of the following criteria as per the PICOS principles: Population: children and adolescents aged from 0 to 19 years as defined by the WHO; Intervention or exposure: children or adolescent classified as obese. Obesity had to be defined using an acceptable objective method, e.g. defined as having a body mass index (BMI) ≥ 95th percentile for children of the same sex and age, or defined as the equivalent of 30 kg/m^2^ International Obesity Task Force (IOTF definition), or defined as obese relative to World Health Organization (WHO) BMI for age and sex charts; Comparison: habitual amount of time spent in MVPA and/or ST of non-obese children and adolescents; Outcomes: habitual amount of time spent in MVPA and/or ST measured by accelerometer and reported in the form of minutes/day of MVPA or ST; MVPA and its relationship to the 60 min/day recommended. All study designs were considered eligible: cross-sectional, longitudinal, case-control studies and intervention studies were eligible if pre-intervention data could be extracted.

### Exclusion criteria

We excluded studies that included only overweight participants, combined overweight and obese groups, or included participants with any known barrier or limitation to physical activity (e.g. physical disability). Studies that used subjective methods, objective (e.g. doubly labelled water) or direct observation methods apart from accelerometer measurements were excluded.

Since the aim of the review was to examine habitual levels of MVPA and ST, studies that measured these variables for less than 6 h per day or over 2 days or less were excluded. Recommendations currently exist for habitual (overall) MVPA rather than MVPA during specific domains (e.g. the after school period) and so studies that focused only on specific periods of the day (e.g. school activity only, or outdoor activity only, or weekend activity only, or weekday activity only, or after-school only) were also excluded. A detailed description of the eligibility criteria is given in [Additional file 1].

### Study selection

**Fig. 1:**
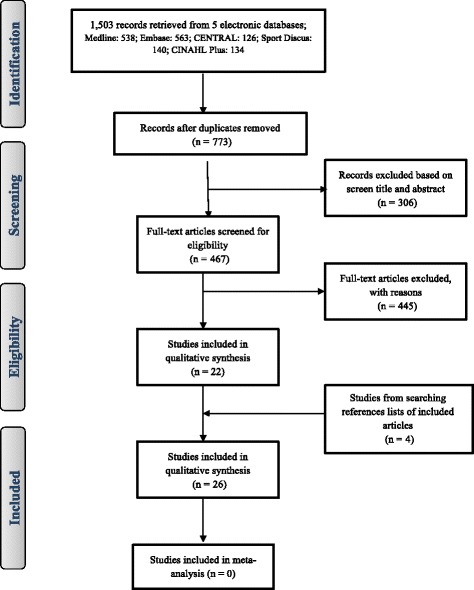
The PRISMA flow diagram with numbers of included and excluded articles at each step of the review process

Titles, abstracts, and full-text articles were screened in duplicate for eligibility by RE and JYP and disagreements were resolved through discussions with other reviewers when required. Reference lists of eligible studies were examined for potentially eligible studies, and studies that cited eligible studies were identified and tested for eligibility. The reviewers were not blinded to authors or journal of publication. Reasons for exclusion are summarised in the study flow diagram (Fig. 1) and available in details from the corresponding author on request.

### Data extraction

A standardised data extraction form was used to populate the evidence tables by RE and repeated by JJR and JYP. The extracted items were: first author, publication year, country, study design, sample group, comparison group-if applicable, accelerometer type, cut points for MVPA and ST, finding of MVPA (minutes/day) and ST (minutes/day or %) data, summary and author conclusions. International recommendations are usually for the achievement of at least 60 min of MVPA every day, but in the eligible studies the achievement of MVPA recommendations was never operationalised in this way. In most studies that referred to the achievement of MVPA recommendations, the mean or median daily MVPA (minutes/day) was provided, and so this was used as a proxy for achievement of recommendations in the present study.

### Data analysis and synthesis

We considered the data for meta-analysis but identified a substantial level of statistical heterogeneity between the studies (I^2^ statistic > 70%) that led to the decision not to present the combined results of individual studies. Hence, we performed a narrative synthesis of the data and present the findings in tabular, textual and graphical form. Data were synthesised by the age and sex of the subgroups as those are factors known to be strongly associated with both the exposure variable, obesity, and the outcomes, MVPA and ST, and so might explain some of the observed findings. The age subgroup was categorised according to the WHO definition of children and adolescence, i.e. as children aged 0 to 9.9 years old and adolescents aged 10.1 to 19 years old. Data for boys, girls and mixed-sex studies are reported separately where possible.

### Quality assessment

Eligible articles were assessed for methodological quality using a 15-item quality assessment scale as shown in [Additional file 2], collapsed to 6 items for scoring, with higher scores suggesting higher study quality. Each eligible study was assessed by RE, and disagreements were resolved by discussion with JJR and JYP. The quality assessment scale was modified from the methodological quality assessment scale of Tooth et al. [23]. This is a reliable and valid tool for assessing the quality of observational studies. It was considered initially for use in its original form, which consists of over 30 items. The modifications to the original scale were made to focus quality assessment on issues of particular importance to accelerometry measurement of physical activity. The modified Tooth et al. tool has been used in several recent systemic reviews of physical activity, all of which have reduced the number of items in the quality assessment to 8 to 17 items, which make up the quality score [24–28].

## Results

### Search results

The PRISMA flow diagram with the numbers of included and excluded articles at each step of the review process is provided in Fig 1. Tables 2 and 3 provide a brief summary of all studies included in this systematic review. Of 1503 papers identified in the initial review of the five databases, 467 were selected for full-text screening and of these, 22 met the inclusion criteria. A further four eligible studies were identified from searching reference of included studies and of previous reviews, giving a total of 26 studies which met the inclusion criteria.

**Table 2:** Overview of relevant characteristics and results of the included studies that involved child participants

Reference^a^	Place &time	Study design	Sample group	Comparison group	Measurement	Accelerometer cut off points	Findings	Meet 60 min/day of MVPA (%) and Author Conclusions
Chung et al. [36]	United States, 2012	Data of NHANES 2003–04 and 2005–06) surveys	n: 95 (47 boys, 48 girls) obese participants with BMI ≥95th to 99th centile	n: 514 (253 boys, 261 girls) participants with BMI ≥ 5th to 85th centile	Actigraph 7164 worn on the right hip for 7 consecutive days during waking hours	Epoch = 1 min	Obese group spent mean of (boys 118 (SD 6) and girls 83 (SD 10) min/day) on MVPA	97, 70% of obese boys and girls respectively achieved an average of 60 min/day of MVPA
			Age: range 6–8 years	Age: range 6–8 years		MVPA using Freedson cut-point (61)	Comparison group spent mean of (boys 129 (SD 4) and girls 104 (SD 4) min/day) on MVPA	98, 90% of the comparison boys and girls respectively achieved an average of 60 min/day MVPA.
								MVPA was significantly lower in obese than comparison group (*p* < 0.05)
Hughes et al. [39]	United Kingdom, 2006	Pairwise comparison study.	n: 53 (25 boys, 28 girls) obese participants with BMI ≥ 98th centile	n: 53 (25boys, 28 girls) participants with BMI < 85th centile.	CSA/MTI, 7164 worn on right hip for 7 consecutive days.	Epoch = 1 min	Obese group spent median of 16 (range 2–72) min/day on MVPA.	Obese participants failed to achieve an average of 60 min/day of MVPA
			“Clinical sample”	Age: mean 8.7 (SD 2.1) years		MVPA > 3200 cpm	Comparison group spent median of 23 (range 7–77) min/day on MVPA.	MVPA was significantly lower in obese than comparison group (*p* < 0.001)
			Age: mean 8.6 (SD 2) years			Sedentary time < 1100 cpm	Obese group spent mean of 81 (SD 7) % of their waking time sedentary.	Sedentary time was similar in obese and comparison group
							Comparison group spent mean of 79 (SD 6) % of their waking time sedentary.	
Hussey et al. [32]	Dublin, 2007	Cross sectional study	n: 7/152 (3 boys, 4 girls) obese participants; with BMI > 97th centile.	n: 121/152 (43 boys, 78 girls) participants with BMI > 75th to 91th centile.	RT3Triaxial accelerometer, worn for 4 days.	Epoch = 1 min	Obese participants spent mean of (boys 14 (95%, CI- 11, 17) and girls 29 (95%, CI- 14, 43) min/day) on MVPA	Obese participants failed to achieve an average of 60 min/day of MVPA
			Age: range 7 to 10 years.	Age: range 7 to 10 years.		MVPA> 3500 cpm	Comparison group spent mean of (boys 39 (95%, CI-33, 45) and girls 24 (95%, CI- 22, 27) min/day) on MVPA	MVPA was significantly lower in obese boys than comparison group (*p* < 0.05)
						Sedentary time cut-points not clearly reported	Obese participants spent mean of (boys 1046 (95%, CI- 934, 1157) and girls 935 (95%, CI- 795, 1075) min/day) sedentary	Sedentary time was significantly higher in obese boys than comparison group (*p* < 0.05)
							Comparison group spent mean of (boys 928 (95%, CI-901, 955) and girls 963 (95%, CI- 941,985) min/day) sedentary	
Maggio et al. [64]	Switzerland, 2010	Cross-sectional study	n: 45/209 obese participants with BMI > 97th centile	n: 85 participants with BMI <90th centile	Actigraph 6471, worn on right hip for 7 consecutive days	Epoch not clearly reported	Obese group spent mean of 60 (SD 3) min/day on MVPA	52% of the obese participants achieved an average of 60 min/day of MVPA
			“Clinical sample”			MVPA > 2000 cpm	Comparison group spent mean of 71 (SD 5) min/day on MVPA	60% of the comparison group achieved an average of 60 min/day MVPA
			Age: mean 9.1 (SD 0.3) years	Age: mean 10 (SD 0.3) years		Sedentary time < 500 cpm	Obese participants spent mean of 71% of their waking time sedentary	MVPA was lower in obese than comparison group but not significantly (*p* = 0.07)
							Comparison group spent mean of 70% of their waking time sedentary	Sedentary time was significantly higher in obese than comparison group (*p* < 0.01)
Metallinos-Katsaras et al. [38]	United States, 2007	Cross sectional study	n: 21 obese children with BMI ≥ 95th centile	n: 35 children; BMI < 95th centile	CSA 7164, worn on the hip for 7 consecutive days	Epoch = 1 min	Obese group spent mean of 269 (SD not given) min/day on MVPA	Obese participants exceeded an average of 60 min/day of MVPA
			Age: range 2 to 5 years	Age: range 2 to 5 years		MVPA cut-points not clearly reported	Comparison group spent mean of 277 (SD not given) min/day on MVPA	MVPA was lower in obese than comparison group but this was not significant (*p* > 0.05)
Thompson et al. [37]	Canada, 2005	Cross sectional study	n: 112 (56 boys, 56 girls) obese participants with BMI ≥ 95th centile	n: 341 (177 boys, 164 girls) participants with BMI ≤ 85th centile	Actigraph 7164, worn on the hip for 7 consecutive days	Epoch = 1 min	Obese group spent mean of (boys 172 (SD 58) and girls 157 (SD 52) min/day) on MVPA	Obese participants exceeded an average of 60 min/day of MVPA
			Age: mean 8 (SD 0.3) years	Age: 3, 7 and 11 years old		MVPA used Freedson cut-point (61)	Comparison group spent mean of (boys 179 (SD 63) and girls 165 (SD 51) min/day) on MVPA	Comparison group exceeded an average of 60 min/day of MVPA
								MVPA was similar in obese and comparison group
Vale et al. [41]	Portugal, 2013	Cross sectional study	n: 59/607 obese children with BMI defined according to the IOTF criteria	n: 425/607 children with BMI defined as non-overweight, non-obese according to the IOTF criteria	ActiGraph GT1M, worn on the hip for 7 consecutive days	Epoch = 5 s	Absolute MVPA not clearly given	MVPA was significantly lower in obese girls than comparison group (*p* < 0.01), but not in boys
			Age: mean 5.1 (SD 0.8) years	Age: range 4–6 years		MVPA ≥1680 cpm		
Wafa et al. [35].	Malaysia, 2014	Case control study.	n: 86 obese participants with BMI ≥ 95th centile	n: 86 participants with BMI < 85th centile matched for age and gender	ActiGraph GT1M, worn for 5 consecutive days	Epoch not reported	Obese group spent median 5 (IQR – 0, 32) min/day on MVPA	Obese participants failed to achieve an average of 60 min/day of MVPA
			Age: median 9.5 (IQR 8,11) years			MVA > 3200 cpm	Comparison group spent of median 9 (IQR – 0, 55) min/day on MVPA	Comparison group failed to achieve an average of 60 min/day of MVPA
						Sedentary time < 1100 cpm	Obese group spent an average of 90% of their waking time sedentary	MVPA was significantly lower in the obese than in the comparison group (*p* < 0.001)
							Comparison group spent an average of 88% of their waking time sedentary	Sedentary time was significantly higher in the obese group than in the comparison group (*p* < 0.001)

*BMI*: body mass index*; cpm*: counts per minutes*; IOTF*: International Obesity Task Force criteria*; MVPA*: moderate- vigorous physical activity; *n*: Number; *S*: Second, Data are expressed as mean, (*SD*) unless otherwise. Freedson *MVPA* cutpoint (61) using the following equation: *METS* = 2.757 + (0.0015 x counts/min) – (0.08957 x age (yr)) – (0.000038 x counts/min x age (yr)).^a^ Studies are listed in alphabetic order

**Table 3:** Overview of relevant characteristics and results of the included studies that involved adolescent participants

Reference^a^	Place & time	Study design	Sample group	Comparison group	Measurement	Accelerometer cut off points	Findings	Meet 60 min/day of MVPA (%) and Author Conclusions
Butte et al. [34]	United States, 2007	Cross sectional study	n: 473 (247 boys, 226 girls) obese participants with BMI ≥ 95th centile	n: 424 (194 boys, 230 girls) participants with BMI <95th centile	Actiwatch, worn on the right hip for 24 h for 3 consecutive days	Epoch = 1 min	Obese spent mean of (boys 88 (SD 50) and girls 74 (SD 46) min/day) on MVPA	62% of all participants (obese and comparison group) achieved an average of 60 min/day of MVPA
			Age: mean 10.8 (SD 3.8) years	Age: mean 10.8 (SD 3.8) years		MVPA and sedentary time cut-points not clearly reported.	Comparison group spent mean of (boys 96 (SD 57) and girls 79 (SD 57) min/day) on MVPA	MVPA was lower in obese than comparison group but not significant
							Obese group spent mean of (boys 357 (SD 118) and girls 345 (SD 122) min/day) sedentary	Sedentary time was significantly higher in obese than comparison group (*P =* 0.001)
							Comparison group spent mean of (boys 305 (SD 121) and girls 308 (SD 131) min/day) sedentary	
Chung et al. [36]	United States, 2012	Data of NHANES 2003–04 and 2005–06) surveys	n: 185 (92 boys, 93 girls) obese participants with BMI ≥95th to 99th centile	n: 987 (489 boys, 498 girls) participants with BMI ≥ 5th to 85th centile	Actigraph 7164 worn on the right hip for 7 consecutive days during waking hours	Epoch = 1 min	Obese group spent mean of (boys 34 (SD 4) and girls 16 (SD 4) min/day) on MVPA	Obese participants failed to achieve an average of 60 min/day of MVPA
			Age: range 12 to 17 years	Age: range 12 to 17 years		MVPA used Freedson cut-point (61)	Comparison group spent mean of (boys 40 (SD 3) and girls 22 (SD 2) min/day) on MVPA	MVPA was significantly lower in obese than comparison group (*p* < 0.05)
Decelis et al. [47]	Malta, 2012	Cross sectional study	n: 34/187 (19 boys, 15 girls) obese participants with BMI defined according to the IOTF criteria	n: 106/187 (53 boys, 53 girls) participants with BMI defined as non-overweight and non-obese according to the IOTF criteria	Actigraph GT3X, worn on right hip for 5 days during waking hours	Epoch not defined	Obese group spent mean of (boys 30 (SD 13) and girls 19 (SD 8) min/day) on MVPA	Obese participants failed to achieve an average of 60 min/day of MVPA
			Age: range 11 to 12 years	Age: range 11 to 12 years		MVPA ≥2912 cpm	Comparison group spent mean of (boys 44 (SD 16) and girls 26 (SD 9) min/day) on MVPA	11% of the comparison group achieved an average of 60 min/day MVPA
						Sedentary time ≤ 727 cpm	Obese group spent mean of (boys 638 (SD 95) and girls 619 (SD 106) min/day) sedentary	MVPA was significantly lower in obese than comparison group (*p* < 0.05)
							Comparison group spent mean of (boys 654 (SD 93) and girls 664 (SD 93) min/day) sedentary	Sedentary time was higher in obese than comparison group, but not significant
Decelis et al. [48]	Malta, 2014	Cross sectional study	n: 113/810 (59 boys, 54 girls) obese participants with BMI defined according to the IOTF criteria	n: 534/810 (254 boys, 280 girls) participants with BMI defined as non-overweight and non-obese according to the IOTF criteria	Actigraph GT3X, worn on right hip for 5 days during waking hours	Epoch = 10 s	Obese group spent mean of (boys 49 (SD 19) and girls 38 (SD 12) min/day) on MVPA	Obese participants failed to achieve an average of 60 min/day of MVPA
			Age: range 10 to 11 years	Age: range 10 to 11 years		MVPA > 2296 cpm	Comparison group spent mean of (boys 61 (SD 22) and girls 44 (SD 15) min/day) on MVPA	MVPA was significantly lower in obese than comparison group (*p* < 0.05)
						Sedentary time < 100 cpm	Obese group spent mean of (boys 553 (SD 94) and girls 610 (SD 125) min/day) sedentary	Sedentary time was higher in obese than comparison group, but not significant
							Comparison group spent mean of (boys 582 (SD 113) and girls 603 (SD 97) min/day) sedentary	
Ekelund et al. [45]	Sweden, 2002	Case control, cross sectional design study.	n: 18 (8 boys, 10 girls) obese participants with BMI defined according to the IOTF criteria	n: 18 (8 boys, 10 girls) participants with BMI defined as non-overweight and non-obese according to the IOTF criteria	CSA 7164, worn on lower part of the back (L 4–5) for 14 days	Epoch = 15 s	Obese group spent mean of (boys 58 (SD 30) and girls 60 (SD 28) min/day) on MVPA	MVPA was significantly lower in obese than comparison group (*p* < 0.05)
			Age: mean (boys 18.1 (SD 1.1), girls 17.3 (SD 1.9) years	Age: mean (boys 18.2 (SD 1.1), girls 17.3 (SD 1.9) years		MVPA cut-points not clearly reported	Comparison group spent mean of (boys 82 (SD 36) and girls 98 (SD 58) min/day) on MVPA	MVPA was similar in obese girls and boys (p < 0.05)
						Sedentary time < 100 cpm	Obese group spent mean of (boys 421 (SD 33) and girls 465 (SD 132) min/day) sedentary	Sedentary time was similar in obese and comparison group
							Comparison group spent mean of (boys 414 (SD 81) and girls 397 (SD 69) min/day) sedentary	
Gyllenham-mer et al. [65]	United States, 2013	Cross sectional study	n: 37 obese girls with BMI ≥ 95th centile	No comparison group	Actigraph GT1M, worn on the right hip for 7 consecutive days	Epoch = 1 min	Obese girls spent mean of 28 (SD 18) min/day on MVPA	Obese girls failed to achieve an average of 60 min/day of MVPA
			Age: mean 15.5 (SD 1.1) years			MVPA cut-points not clearly reported	Obese participants spent 63% (SD 7) of their waking time sedentary	MVPA was lower in obese girls compared to published data from healthy adolescents
						Sedentary time = 100 cpm		
Kitzman-Ulrich et al. [31]	United States, 2010	Randomized trial ACT (Active by Choice Today) data at baseline used here	n: 242/669 (98 boys, 144 girls) obese participants with BMI > 95th centile	n: 314/ 669 (138 boys, 176 girls) participants with BMI < 85th centile	Actical, worn on the right hip for 7 days all the day	Epoch = 1 min	Obese participants spent mean of (boys 46 (SD 20) and girls 36 (SD 15) min/day) on MVPA	Obese participants failed to achieve an average of 60 min/day of MVPA
			Age: mean 11.4 (SD 0.7) years	Age: mean 11.4 (SD 0.7) years		MVPA and sedentary time cut-points not clearly reported	Comparison group spent mean of (boys 65 (SD 27) and girls 46 (SD 20) min/day) on MVPA	MVPA level was significantly lower in obese than comparison group (p < 0.05)
Maggio et al. [64]	Switzerland, 2014	Case control study	n: 24 (12 boys, 12 girls) obese participants with BMI ≥ 97th centile. “Clinical sample”	n: 25 (12 boys, 13 girls) participants with BMI <90th centile	Actigraph GT1M, worn for at least 4 days	Epoch and MVPA cut-points not clearly reported	Obese participants spent mean of 43 (SD 19) min/day on MVPA	Obese participants failed to achieve an average of 60 min/day of MVPA
			Age: mean 13.9 (SD 1.2) years	Age: mean 13.2 (SD 1.7) years		Sedentary time < 500 cpm	Comparison group spent mean of 58 (SD 30) min/day on MVPA	MVPA was similar in obese girls and boys (p < 0.05)
								MVPA was significantly lower in obese than comparison group (*P =* 0.01)
Martins et al. [66]	Portugal, 2015	Cross sectional baseline study	n: 131 (48 boys, 83 girls) obese participants with BMI defined according to the IOTF criteria	No comparison group	Actigraph GT3x, worn for 7 consecutive days.	Epoch = 1 mim	Obese participants spent mean of (boys 65 (SD 28) and girls 51 (SD 22) min/day) on MVPA	Obese participants failed to achieve an average of 60 min/day of MVPA
			Age: mean 10.3 (SD 3.6) years			MVPA cut-points not clearly reported	Participants spent mean of (boys 575 (SD 108) and girls 562, (SD 82) min/day) sedentary	MVPA was significantly lower in obese girls than obese boys (p < 0.05)
						Sedentary time = 0–100 cpm		MVPA was lower in obese participants compared to published data from healthy children and adolescents
McMurray et al. [67]	United States, 2008	Baseline data of Randomized controlled TAAG “Trial of Activity for Adolescent Girls”	n: 184/1021 obese girls with BMI ≥ 95th centile.	n: 645/1021 participants with BMI < 85th centile	Actigraph MTI, worn for 6 consecutive days	Epoch = 30 s	Obese girls spent mean of 21 (SD 2) min/day on MVPA	Obese participants failed to achieve an average of 60 min/day of MVPA
			Age: range 11 to 14 years	Age: range 11 to 14 years		MVPA cut-points not clearly reported.	Comparison group spent mean of 25 (SD 1) min/day on MVPA	MVPA was significantly lower in the obese than the comparison group (p = 0.01)
Page et al. [43]	United Kingdom, 2005	Cross sectional study	n: 25 (14 boys, 11 girls) obese participants with BMI ≥ 99th centile. “Clinical and non clinical sample”	n: 108 (54 boys, 54 girls) participants with BMI < 99th centile	Actigraph 7164, worn on the waist for 7 consecutive days	Epoch and MVPA cut-points not clearly reported.	Obese participants spent mean of (boys 140 (SD 47) and girls 105(SD 48) min/day) on MVPA	Obese participants exceeded an average of 60 min/day of MVPA
			Age: mean 10.5 (SD 0.8) years				Comparison group spent mean of (boys 176 (SD 52) and girls 149 (SD 52) min/day) on MVPA.	MVPA was significantly lower in obese compared to comparison group (*p* = 0.02)
Peart et al. [68]	United States, 2011	Combined data of cross sectional NHANES (2003–04, 2005–06) surveys	n: 434/2368 (217 boys, 217 girls) obese participants with BMI ≥ 95 centile	n: 1469/ 2368 (749 boys, 720 girls) participants with BMI < 85th centile	Actigraph 7164, worn on hip over 7 day	Epoch = 1 min	Obese participants spent mean 28 (SD 35) min/day on MVPA	Obese participants failed to achieve an average of 60 min/day of MVPA
			Age: mean 15.4 (SD 2.2) years	Age: mean 15.4 (SD 2.2) years		MVPA≥1500 cpm	Comparison group spent mean of 32 (SD 29) min/day on MVPA	MVPA was similar in obese boys and girls
								MVPA was lower in obese than comparison group but not significantly (p > 0.05)
Ruiz et al. [46]	10 centers in 9 European countries, 2011	Cross sectional study	n: 104/2200 (45 boys, 59 girls) obese participants with BMI ≥ 95th centile	n: 1592/2200 (870 boys, 722 girls) participants with BMI < 85th centile	Actigraph GT1M, worn lower back for 7 consecutive days	Epoch = 15 s	Obese participants spent mean of (boys 60 (95%, CI- 53, 68) and girls 44 (95%, CI- 38,50) min/day) on MVPA	MVPA was significantly lower in obese boys than comparison boys group (*p* = 0.002)
			Age: median 14.9 (IQR 12.8 to 15.8) years	Age: median 14.9 (IQR 12.8 to 15.8) years		MVPA ≥2000 cpm	Comparison group spent mean of (boys 67, (95%, CI- 65, 69) and girls 51 (95%, CI- 49,52) min/day) on MVPA	Sedentary time was significantly higher in obese girls than comparison girls group (*p* = 0.006)
						Sedentary time < 100	Obese participants spent of (boys 68% and girls 71%) of their waking time sedentary	
							Comparison group spent mean of (boys 69% and girls 72%) of their waking time sedentary	
Shoup et al. [69]	United States, 2008	Cross sectional study	n: 85 obese participants; BMI ≥ 99th centile	n: 92 participants with BMI ≥85th and <95th centile matched for age	Actigraph 7164, worn on waist for 7 consecutive days.	Epoch and MVPA cut-points not clearly reported.	Obese participants spent mean of 54 (SD 22) min/day on MVPA.	40% (*n* = 34) of the obese group achieved an average of 60 min/day on MVPA
			“Clinical sample”				Comparison group spent mean of 59 (SD 30) min/day on MVPA.	40% (n = 35) of the comparison group achieved 60 min/day of MVPA
			Age: mean 10.6 (SD 1.4) years					MVPA was similar in obese and comparison group
St George et al. [29].	United States, 2013	Baseline data of ACT Randomized trial (Active by Choice Today).	n: 484/1422 (203 boys, 281 girls) obese participants with BMI ≥ 95th centile.	n: 684/1422 (321 boys, 363 girls) participants with BMI < 85th centile.	Actical, worn on the right hip for 7 consecutive days.	Epoch = 1 min	Obese participants spent mean of 37 (SD 22) min/day on MVPA.	Obese participants failed to achieve an average of 60 min/day of MVPA
			Age: mean 11.3 (SD 0.6) years	Age: mean 11.4 (SD 0.6) years		MVPA used Puyau cut-point (66).	Comparison group spent mean of 47 (SD 28) min/day on MVPA.	MVPA was significantly lower in obese than comparison group (p < 0.01)
Starkoff et al [30].	United States, 2014	Cross sectional study.	n: 16 (5 boys, 11 girls) obese participants with BMI ≥ 95 centile.	No comparison group	Actical, worn on the right hip for 5 days during waking time	Epoch = 1 min	Obese participants spent mean of (boys 26 (SD 36) and girls 19 (SD 17) min/day) on MVPA	12.5% of the obese group achieved an average of 60 min/day of MVPA
			“Clinical sample”			Sedentary time and MVPA cut-points not clearly reported	Obese participants spent mean of (boys 731 (SD 110) and girls 726 (SD 98) min/day) on sedentary	MVPA was lower in obese participants compared to published data from healthy adolescents
			Age: mean 14.8 (SD 1.5) years.					
Thompson et al. [36].	Canada, 2005	Cross sectional study.	n: 171 (93 boys, 78 girls) participants with BMI ≥ 95th centile.	n: 716 (327 boys, 389 girls) participants with BMI ≤ 85th centile.	Actigraph 7164, worn on the hip for 7 consecutive days.	Epoch = 1 min	Obese group spent mean of (boys 53 (SD 26) and girls 48 (SD 25) min/day) on MVPA.	Obese participants failed to achieve an average of 60 min/day of MVPA
			Age: range 12 to 16 years	Age: range 12 to 16 years		MVPA used Freedson cut-point (61)	Comparison group spent mean of (boys 58 (SD 30) and girls 47 (SD 24) min/day) on MVPA.	Comparison group failed to achieve an average of 60 min/day of MVPA
								MVPA was similar in obese and comparison group
Trost et al. [44].	United States, 2001	Cross sectional study.	n: 54 obese participants with BMI ≥ 95th centile.	n: 133 non obese with	CSA 7164, worn on right hip for 7 consecutive days.	Epoch = 1 min	Obese participants spent mean of 70 (SD 6) min/day on MVPA.	Obese participants achieved an average of 60 min/day of MVPA
			Age: mean 11.4 (SD 0.6) years.	BMI < 95th centile.		MVPA used Freedson cut-point (61)	Comparison group spent mean of 82 (SD 4) min/day on MVPA.	Comparison group achieved an average of 60 min/day of MVPA
				Age: mean 11.4 (SD 0.6) years.				MVPA was significantly lower in obese than comparison group (p < 0.001)
Vanhelst et al. [33].	France, 2013	Cross sectional study.	n: 56 obese participants with BMI ≥ 97th centile.	No comparison group	RT3 worn on right hip up to 21 consecutive days.	Epoch = 1 min	Participants spent mean of 22 (SD 12) min/day on MVPA.	Obese participants failed to achieve an average of 60 min/day of MVPA
			“Clinical sample”			MVPA cut-points not clearly reported.		MVPA was lower in obese participants compared to published data from healthy children and adolescents
			Age: mean 12.8 (SD 2.9) years.			Sedentary time = 0–40 cpm		
Wang et al. [42]	Chine, 2013	Cross sectional “large-scale study”	n: 175/2163 (115 boys, 60 girls) obese participants with BMI defined according to the IOTF criteria.	n: 1709/2163; 808 boys; 901 girls with BMI defined as non-overweight, non-obese according to the IOTF criteria	ActiGraph GT3X, worn on right hip for 7 consecutive days	Epoch = 1 min	Obese group spent mean of (boys29 (SD 18) and girls 24 (SD 13) min/day) on MVPA	7% of the obese boys achieved an average of 60 min/day of MVPA
			Age: mean 13.41 (SD 2.25) years.	Age: mean 13.41 (SD 2.25) years.		MVPA cut-points not clearly reported	Comparison group spent mean of (boys 35(SD 19) and girls 22 (SD 14) min/day) on MVPA	10%, 2% of the comparison boys and girls respectively achieved an average of 60 min/day of MVPA
						Sedentary time < 100 cpm	Obese group spent mean of (boys 480 (SD 107) and girls 490 (SD 89) min/day) sedentary	MVPA was similar in obese and comparison groups
							Comparison group spent mean of (boys 521 (SD 113) and girls 533, (SD 103) min/day) sedentary	Sedentary time was similar in obese and comparison groups

*BMI*: body mass index; cpm: counts per minutes; *IOTF*: International Obesity Task Force criteria; *MVPA*: moderate- vigorous physical activity; *n*: Number; *S*: Second, Data are expressed as mean, (*SD*) unless otherwise. Freedson *MVPA* cutpoint (61) using the following equation: *METS* = 2.757 + (0.0015 x counts/min) – (0.08957 x age (yr)) – (0.000038 x counts/min x age (yr)). ^a^Studies are listed in alphabetic order

### Studies characteristics

Of the 26 included studies: six studies involved children, 18 studies involved adolescents and two studies involved both children and adolescents. Further, 22/26 compared MVPA data in those with obesity with a non-obese peers, while 13/26 studies also provided data on accelerometer measured ST; 10/13 studies compared ST data in those with obesity with non-obese peers.

### Measurement protocol

The ActiGraph was the most common accelerometer type used to measure habitual MVPA and/or ST, used in 20/26 studies, though with a variety of different ActiGraph models and approaches to data collection and reduction. Of the remaining six studies: three used the Actical accelerometer [29–31]; two the Triaxial Research Tracker (RT3) accelerometer [32, 33]; and one the Actiwatch accelerometer [34].

### MVPA and ST in obese children

Eight eligible studies involved obese children, with a total sample size of 2138 children (478 with obesity; 131 boys, 136 girls and 211 no sex specified). Two of the eligible studies were clinical samples with study participants recruited from outpatient clinics. Eligible studies were from different nations with one study from Asia [35], three from Canada and USA [36–38] and four from Europe [32, 39–41], with the study characteristics summarized in Table 2. In four studies, MVPA data of boys and girls were reported separately while in the other four studies MVPA data were reported as mixed sex. 7/8 of eligible studies reported mean daily time spent in MVPA in minutes; in four studies mean time spent in MVPA was < 60 min/day. Furthermore, in 2/7 of the eligible studies, children with obesity reached or exceeded 60 min of MVPA per day [37, 38], while in one study they came close to a mean of 60 min/day of MVPA [36]. In all cases time spent in MVPA in the children who were obese was compared to the comparison group (non-obese peers). In only one study was the mean time spent in MVPA similar in both groups [37]; in three studies, time spent in MVPA was significant lower in children with obesity than in the comparison group [35, 36, 39], while in two studies time spent in MVPA of children with obesity was lower than the comparison group but differences were not significant [38, 40]. In the other 2 studies, time spent in MVPA of children with obesity was different in terms of gender compared to the comparison group: Hussey et al. reported that mean MVPA was significantly lower in boys with obesity but not in girls [32]; while Vale et al. reported that mean time spent in MVPA was significantly lower in girls with obesity but not in boys [41] compared to the comparison groups.

With respect to ST, 4/8 eligible studies reported on accelerometer-measured time spent in SB of children with obesity with a total sample size of 536 children (191 with obesity; 28 boys, 32 girls and 131 no sex specified). In one study, ST data of boys and girls was reported separately while in other the data were reported as mixed sex. Across all four eligible studies, mean time spent in SB was > 70% of waking time [32, 35, 39, 40]. In 3/4 of the studies ST was significantly higher in the obese than the non-obese groups, although, in one study it was significantly higher in boys with obesity but not in girls [32]. In one study ST was similar in both groups [39].

### MVPA and ST in obese adolescents

Twenty of the eligible studies involved adolescents, with a total sample size of 12,601 adolescents (3045 with obesity; 1615 boys, 1575 girls and 195 no sex specified). Four of the eligible studies were clinical samples with participants recruited from outpatient clinics. Eligible studies were from different nations with one study from Asia [42], 11 from Canada and the USA, and eight from Europe, with the study characteristics summarized in Table 3. In 12/20 studies, MVPA data of boys and girls were reported separately; in 6/20 studies MVPA data were reported as mixed sex, while the other two studies involved only adolescent girls. All 20 eligible studies reported mean daily time spent in MVPA in minutes and in these studies it ranged from a low of 16 (SD 4) minutes/day [36] to a high of 140 (SD 47) minutes/day [43]. In only 2/ 20 studies did daily time spent in MVPA reach an average of at least 60 min [43, 44] in the adolescents who were obese. A total of 16/20 eligible studies compared time spent in MVPA of those with obesity with a comparison group: in 3/16 time spent in MVPA was similar between obese and non-obese groups, while in 10/16 mean time spent in MVPA was significantly lower in adolescents with obesity than in non-obese peers.

In regard to time spent in SB, nine out of the 20 eligible studies reported on accelerometer measured ST in adolescents with obesity with a total sample size of 5484 adolescents (1101 with obesity; 546 boys and 555 girls), as summarised in Table 3. In 8/9 studies, ST data of boys and girls were reported separately and 1/9 study involved only adolescent girls. In 7/9 studies, mean daily ST was reported in minutes and in these studies it ranged from a low of 345 (SD 122) minutes/day [34] to a high of 731 (SD 110) minutes/day [30]. In 6/9 studies there was a comparison group; in 2/6 studies mean daily ST was similar in obese and non-obese groups [42, 45]; in 2/6 studies ST was significantly higher in those with obesity than in the non-obese comparison groups [34, 46], while in the other 2/6 studies it was higher in the adolescents with obesity, but not significantly so [47, 48].

**Fig. 2:**
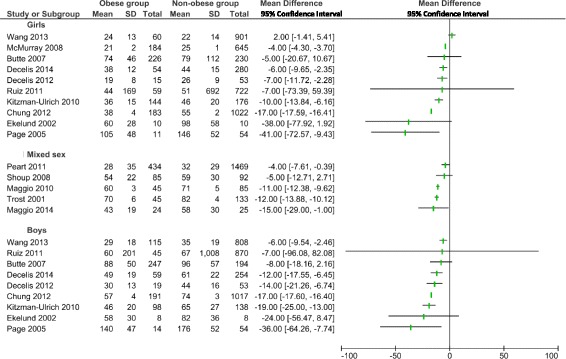
Forest plot of the comparison of moderate-to-vigorous intensity physical activity between children and adolescents with obesity and non-obese participants by sex. SD: standard deviation; CI: 95% Confidence interval

**Fig. 3:**
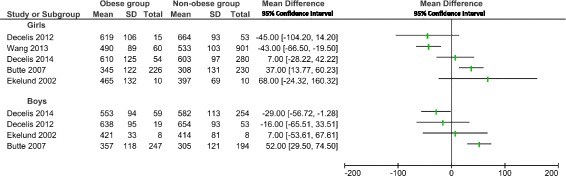
Forest plot of the comparison of sedentary time between children and adolescents with obesity and non-obese participants by sex. SD: standard deviation; CI: 95% Confidence interval

A graphical synthesis of the mean differences and 95% CI of time spent in MVPA by sex for both children and adolescents with obesity and non-obese groups, is shown in Fig. 2. A summary of the mean differences and 95% CI of time spent in SB by sex for both children and adolescents with obesity and non-obese groups, is shown in Fig. 3.

### Study quality assessment

Study quality assessment summaries are given in [Additional file 3]. One study scored 4/6 12 studies scored 5/6 while 13 scored 6/6 on study quality.

## Discussion

This systematic review provided clear evidence that children and adolescents with obesity have lower than the recommended levels of MVPA. In most of the eligible studies, daily time spent in MVPA averaged less than the 60 min/day recommended in many guidelines. When comparing MVPA level between obese and non-obese peers, the findings indicated that daily MVPA was lower in children and adolescents with obesity.

In reviewing the methodology of the studies, it is noteworthy that the precise accelerometer methodology was often not stated clearly, or sometimes not stated at all, in the eligible studies particularly with respect to the cut-off used to define MVPA (Tables 2 and 3). However, mean time spent in MVPA was broadly similar within studies that employed comparable accelerometer methods. For example, in the two eligible Actigraph studies which used a cut-off of 2000 counts per minutes (cpm) to define MVPA (Maggio et al. [40], age 4–17 years old, and Ruiz et al. [46], age 12–17 year olds) mean daily time spent in MVPA was 60 min/day in the boys with obesity in the sample studied by Ruiz et al. [46], and 60 min/day in the boys with obesity studied by Maggio et al. [40]. Both of these studies might suggest the tentative conclusion that time spent in MVPA is relatively high in adolescents who are obese, possibly suggesting that adolescence and/or obesity do not present major barriers to MVPA. In contrast, three of the eligible Actigraph studies used higher MVPA cut-offs which ranged between 2912 cpm in 11–12 year olds: Decelis et al. [47] and 3200 cpm Hughes et al. [39] and Wafa et al. [35] in 8 and 9 year olds respectively. These studies found that mean daily time spent in MVPA was 30 min in boys and 19 min in girls with obesity [47] and a median of 16 min for both sexes combined in the study by Hughes et al. [39] and 5 min/day in the study by Wafa et al. [35]. The majority of children and adolescents with obesity achieved means of < 30 min of daily time spent in MVPA in studies with cut-offs of ≥2912 cpm [32, 38, 39, 43, 44].

Further, it should be noted that recommendations for MVPA state that 60 min per day is a minimum every day (e.g. usually operationalized as 7 days in a week) [49–51], but adherence to recommendations was never operationalized in this way in any of the 26 eligible studies. We therefore used a mean or median daily MVPA of 60 min as a proxy for adherence, though this is conservative because in many individuals where 60 min MVPA/day was reached as an average, time spent in MVPA would have fallen below 60 min/day on at least one of the monitored days.

With respect to sedentary time, the present review found that studies fairly consistently reported that children and adolescents with obesity accumulated a high amount of ST during their waking hours, ranging typically between 65 and 90% of their waking monitoring time: 10 h was the mean daily ST in all 13/26 studies, which reaches or exceeds typical Actigraph measured levels of ST in North-American adults from surveys such as NHANES. All eligible studies, which had comparison groups, found ST was fairly consistent with no marked differences between obese and non-obese peers.

In the present systematic review, the level of heterogeneity between eligible studies made it impossible to combine data in a formal meta-analysis. The heterogeneity noted was due to differences in the location of the studies, differences in the way obesity was defined (different BMI cut-off points and different reference data), or differences in accelerometer models and methodology. Therefore, we narratively synthesized the differences in the time spent in MVPA and ST between obese and control groups by age and sex. Additionally, future research would benefit from an attempt to obtain the original study participant data and to reanalyse that with a common methodology, though this was beyond the scope of the present study.

### Comparisons with other studies

We believe that the present study is the first systematic review to ask whether or not levels of accelerometer measured MVPA are adequate in children and adolescents with obesity, and whether time spent in MVPA and ST differed between obese and comparison groups based on accelerometer data. There are therefore no directly comparable studies. However, our findings are consistent with some studies on the correlates and determinants of objectively MVPA [52, 53], and consistent with a growing belief that obesity is associated with reduced MVPA and that low MVPA could be both a cause of obesity and a consequence of obesity, i.e., “bidirectional causation” [20, 54, 55].

### Review and evidence strengths and weaknesses

The evidence considered by our review had a number of strengths. Firstly, it investigated the accelerometer-measured time spent in MVPA and ST of children and adolescents with obesity, with clear definitions of obesity so that samples included in the review were not contaminated by the inclusion of overweight but non-obese individuals. Secondly, there are several methodological strengths to this study. Studies were identified from an extensive search of the published literature conducted in a range of databases, over the last 15 years, covering the time when accelerometers started to become available and popular in PA research and, more recently, sedentary behavior research. The broad definition of search terms applied across multiple databases enabled the searching and identification across many potential studies with no limitations on place of publication, sample size or country of origin. Restricting eligibility to studies using accelerometry was important in increasing confidence in the measurement of MVPA [19, 56–58]. The included studies were in general rated as being of high or very high methodological quality with respect to their accelerometry methods. Also, in some cases the eligible studies were based on large nationally representative samples or surveys another strength in terms of generalizability.

There were some sources of weakness in our systematic review. Firstly, since studies had to be published in peer-reviewed journals in English, this may have excluded some relevant evidence. The generalisability of review results is subject to certain limitations; for instance, eligible studies in our systematic review were from high-income nations, and we lacked data from low-middle income countries. Most of the included studies were based on relatively small samples of obese children and adolescents with a total (*n* = 14,739 participants; *n* = 3523 with obesity) and their power to estimate habitual MVPA might have been limited, and thus the extent to which the results observed are generalizable to the general obese paediatric population is unclear. The method of quality assessment in our review, in which the original 15 items scale has been collapsed to 6 items, has been used in several accelerometry systematic reviews [24–28]. However, the process of collapsing 15 items to a six-item might have reduced the possibility of identifying differences in quality between studies.

Eligible studies generally obtained MVPA and ST data using the ActiGraph accelerometer, but methods used varied between studies. Methodological differences include: the definition of epoch, the number of hours and days of data constituting a valid/acceptable data set, MVPA and ST cut-points, and the choice of non-wear criteria. These methodological variation tends to produce meaningful differences in MVPA and ST estimates between studies [16] and also make it difficult to compare across studies. However, while there were multiple differences between studies in accelerometry methodology (e.g. in epochs, cut-points, handling of non-wear time, duration of accelerometry monitoring), in all cases the methods were the same within studies between the obese and non-obese comparison groups, so these methodological differences probably had limited effect on the ability of studies to identify differences in MVPA and ST between the obese and non-obese. Finally, the validity of accelerometry (in particular hip-worn accelerometry, the method in almost all eligible studies) to determine ST is less well established than the validity of this placement for measurement of MVPA [59]. Hip-worn accelerometers are not designed to measure posture, and devices such as inclinometers may provide improved measurement. Accelerometers are used widely to measure ST though, and there is some evidence of validity for group-comparisons as here (obese vs non-obese comparisons) [19, 16].

## Conclusions

In summary, the data presented in our review demonstrated that a high percentage of obese children and adolescents did not achieve the minimum amount of 60 min per day MVPA that is recommended in guidelines and tended to spend what appeared to be the vast majority of their waking hours sedentary. Children and adolescents with obesity were generally slightly less physically active and slightly more sedentary compared to comparison groups, though the present review cannot test whether they were less active or more sedentary before becoming obese.

Given the many and varied health and non-health benefits of MVPA in children and youth [60, 61], and emerging evidence that ST influences health outcomes in children and adolescents [62, 63] the present review highlights the need to focus on increasing MVPA and reducing ST among children and adolescents with obesity, and the importance of raising these issues in clinical settings as part of treatment for obesity. Treatment of childhood and adolescent obesity should clearly involve a focus on increasing MVPA and reducing ST as recommended in multiple evidence based treatment and prevention guidelines published in recent years.

## Additional files

Additional file 1: Inclusion and exclusion criteria for selection of studies. (XLSX 9 kb) MVPA: Moderate-to-Vigorous Intensity Physical Activity; PA: physical activity; SB: sedentary behavior. (XLSX 9 kb)

Additional file 2: Study Quality Assessment Criteria, modified from Tooth et al. (22). MVPA: Moderate-to-Vigorous Intensity Physical Activity (XLSX 34 kb)

Additional file 3: Methodological quality assessment of the included studies. (XLSX 26 kb)

## Abbreviations

BMI: Body mass index
MVPA: Moderate-to-vigorous physical activity
PA: Physical activity
SB: Sedentary behavior
ST: Sedentary Time

## References

[1] Caballero B (2007). The global epidemic of obesity: an overview. Epidemiol Rev.

[2] Lobstein T (2004). Obesity in children and young people: a crisis in public health. Obes Rev.

[3] Dietz WH (1998). Health consequences of obesity in youth: childhood predictors of adult disease. Pediatrics.

[4] Waters E (2014). Interventions for preventing obesity in children. Sao Paulo Med J.

[5] Hardy LL (2012). Co-occurrence of obesogenic risk factors among adolescents. J Adolesc Health.

[6] Eissa MA, Gunner KB, University of Texas-Houston Health Science center (2004). Evaluation and management of obesity in children and adolescents. J Pediatr Health Care.

[7] Leech RM, McNaughton SA, Timperio A. The clustering of diet, physical activity and sedentary behavior in children and adolescents: a review. Int J Behav Nutr Phys Act. 2014;11(4). doi:10.1186/1479-5868-11-4. 10.1186/1479-5868-11-4 PMC3904164 24450617

[8] Rippe JM, Hess S (1998). The role of physical activity in the prevention and management of obesity. J Am Diet Assoc.

[9] Mitchell JA, Byun W (2014). Sedentary behavior and health outcomes in children and adolescents. Am J Lifestyle Med.

[10] Simon C (2008). Successful overweight prevention in adolescents by increasing physical activity: a 4-year randomized controlled intervention. Int J Obes.

[11] Stroebele N, Hill JO, Willich SN (2011). Identifying the energy gap in the German population using results from representative national health surveys (1985–2002). Public Health Nutr.

[12] Lau DC (2007). 2006 Canadian clinical practice guidelines on the management and prevention of obesity in adults and children [summary]. CMAJ.

[13] Organization WH. Global recommendations on physical activity for health; 2010. 26180873

[14] Tremblay MS (2011). Canadian sedentary behaviour guidelines for children and youth. Appl Physiol Nutr Metab.

[15] Tremblay MS (2011). New Canadian physical activity guidelines. App Phys Nut Meta-Phys Appli Nutr Et Meta.

[16] Reilly JJ (2008). Objective measurement of physical activity and sedentary behaviour: review with new data. Arch Dis Child.

[17] Riddoch CJ (2007). Objective measurement of levels and patterns of physical activity. Arch Dis Child.

[18] Reilly JJ (2010). Low levels of objectively measured physical activity in preschoolers in child care. Med Sci Sports Exerc.

[19] Pate RR, O'Neill JR, Mitchell J (2010). Measurement of physical activity in preschool children. Med Sci Sports Exerc.

[20] Bauman AE (2012). Correlates of physical activity: why are some people physically active and others not?. Lancet.

[21] Pate RR (1995). Physical activity and public health. A recommendation from the Centers for Disease Control and Prevention and the American College of Sports Medicine. JAMA.

[22] Moher D (2009). Preferred reporting items for systematic reviews and meta-analyses: the PRISMA statement. Open Med.

[23] Tooth L (2005). Quality of reporting of observational longitudinal research. Am J Epidemiol.

[24] Reilly JJ, et al. Contribution of school recess to daily physical aactivity: systematic review. Health Behav Pol Rev. 2016;3(6):581–589(9).

[25] Martin A, et al. Contribution of walking to school to individual and population moderate-vigorous intensity physical activity: systematic review and meta-analysis. Pediatr Exerc Sci. 2016;28(3):353–63. 10.1123/pes.2015-0207 26882871

[26] Jones RA (2013). Tracking physical activity and sedentary behavior in childhood a systematic review. Am J Prev Med.

[27] Tanaka C, Reilly JJ, Huang WY (2014). Longitudinal changes in objectively measured sedentary behaviour and their relationship with adiposity in children and adolescents: systematic review and evidence appraisal. Obes Rev.

[28] Elmesmari R (2017). Accelerometer measured levels of moderate-to-vigorous intensity physical activity and sedentary time in children and adolescents with chronic disease: a systematic review and meta-analysis. PLoS One.

[29] St George SM (2013). Weight status as a moderator of the relationship between motivation, emotional social support, and physical activity in underserved adolescents. J Pediatr Psychol.

[30] Starkoff BE (2014). Sedentary and physical activity habits of obese adolescents. Am J Health Educ.

[31] Kitzman-Ulrich H (2010). Relationship of body mass index and psychosocial factors on physical activity in underserved adolescent boys and girls. Health Psychol.

[32] Hussey J (2007). Relationship between the intensity of physical activity, inactivity, cardiorespiratory fitness and body composition in 7-10-year-old Dublin children. Br J Sports Med.

[33] Vanhelst J (2013). Concurrent validity of the modified international physical activity questionnaire for French obese adolescents. Percept Motor Skills.

[34] Butte NF (2007). Physical activity in nonoverweight and overweight Hispanic children and adolescents. Med Sci Sports Exerc.

[35] Wafa SW (2014). Objectively measured habitual physical activity and sedentary behaviour in obese and non-obese Malaysian children. J Trop Pediatr.

[36] Chung AE (2012). Physical activity and BMI in a nationally representative sample of children and adolescents. Clin Pediatr.

[37] Thompson AM (2009). Are overweight students in grades 3, 7, and 11 less physically active than their healthy weight counterparts?. Int J Pediatr Obes.

[38] Metallinos-Katsaras ES (2007). The association between an objective measure of physical activity and weight status in preschoolers. Obes.

[39] Hughes AR (2006). Habitual physical activity and sedentary behaviour in a clinical sample of obese children. Int J Obes.

[40] Maggio AB (2010). Reduced physical activity level and cardiorespiratory fitness in children with chronic diseases. Eur J Pediatr.

[41] Vale S (2013). Physical activity guidelines and preschooler's obesity status. Int J Obes.

[42] Wang C, Chen P, Zhuang J (2013). A national survey of physical activity and sedentary behavior of Chinese city children and youth using accelerometers. Res Quar Exer S.

[43] Page A (2005). Physical activity patterns in nonobese and obese children assessed using minute-by-minute accelerometry. Int J Obes.

[44] Trost SG (2001). Physical activity and determinants of physical activity in obese and non-obese children. Int J Obes Rel Met Dis J Int Assoc Stud Obes.

[45] Ekelund U (2002). Physical activity but not energy expenditure is reduced in obese adolescents: a case-control study. Am J Clin Nutr.

[46] Ruiz JR (2011). Objectively measured physical activity and sedentary time in European adolescents the HELENA study. Am J Epidemiol.

[47] Decelis A, Jago R, Fox KR (2014). Objectively assessed physical activity and weight status in Maltese 11-12 year-olds. Euro J Sport Sci EJSS : Offic J Euro Col Sport Sci.

[48] Decelis A, Jago R, Fox KR (2014). Physical activity, screen time and obesity status in a nationally representative sample of Maltese youth with international comparisons. BMC Public Health.

[49] Organization, W.H (2010). Global recommendations on physical activity for health.

[50] Twisk JW (2001). Physical activity guidelines for children and adolescents: a critical review. Sports Med.

[51] Barlow SE, Dietz WH (1998). Obesity evaluation and treatment: expert committee recommendations. Mater Child Health Bureau, Health Res Ser Admin Dep Health Human Ser Pediatrics.

[52] Ekelund U (2012). Moderate to vigorous physical activity and sedentary time and Cardiometabolic risk factors in children and adolescents. Jama-J Am Med Assoc.

[53] Jimenez-Pavon D, Kelly J, Reilly JJ (2010). Associations between objectively measured habitual physical activity and adiposity in children and adolescents: systematic review. Int J Pediatr Obes.

[54] Must A, Tybor DJ (2005). Physical activity and sedentary behavior: a review of longitudinal studies of weight and adiposity in youth. Int J Obes.

[55] Richmond RC (2014). Assessing causality in the association between child adiposity and physical activity levels: a Mendelian randomization analysis. PLoS Med.

[56] Toschke JA (2007). Reliability of physical activity measures from accelerometry among preschoolers in free-living conditions. Clin Nutr.

[57] Nyberg G, Ekelund U, Marcus C (2009). Physical activity in children measured by accelerometry: stability over time. Scand J Med Sci Sports.

[58] Bender JM (2005). Children's physical activity: using accelerometers to validate a parent proxy record. Med Sci Sports Exerc.

[59] van Nassau F (2015). Validity and responsiveness of four measures of occupational sitting and standing. Int J Behav Nutr Phys Act.

[60] Janssen I, LeBlanc AG. Systematic review of the health benefits of physical activity and fitness in school-aged children and youth. Int J Behav Nutr Phys Act. 2010;7(40). doi:10.1186/1479-5868-7-40. 10.1186/1479-5868-7-40 PMC2885312 20459784

[61] Strong WB (2005). Evidence based physical activity for school-age youth. J Pediatr.

[62] Mann K (2017). Longitudinal study of the associations between change in sedentary behavior and change in adiposity during childhood and adolescence: Gateshead millennium study. Int J Obes (2005).

[63] Belcher BR (2015). Effects of interrupting children's sedentary behaviors with activity on metabolic function: a randomized trial. J Clin Endocrin Metabol.

[64] Maggio ABR, et al. High bone density in adolescents with obesity is related to fat mass and serum leptin concentrations. J Pediatr Gastroenterol Nutr. 2014;58(6):723–8. 10.1097/MPG.0000000000000297 24399210

[65] Gyllenhammer LE, et al. Objective habitual physical activity and estradiol levels in obese Latina adolescents. J Phys Act Health. 2013;10(5):727–33. 10.1123/jpah.10.5.727 PMC3779056 23038707

[66] Martins C, et al. Physical Activity is related to Fatty Liver Marker in Obese Youth, Independently of Central Obesity or Cardiorespiratory Fitness. Journal of Sports Science & Medicine. 2015;14(1):103–9. PMC4306761 25729297

[67] McMurray RG, et al. Do overweight girls overreport physical activity? Am J Health Behav. 2008;32(5):538–46. 10.5555/ajhb.2008.32.5.538 PMC2430385 18241138

[68] Peart T, et al. Weight Status in US Youth: The Role of Activity, Diet, and Sedentary Behaviors. Am J Health Behav. 2011;35(6):756–64. 10.5993/ajhb.35.6.11 22251766

[69] Shoup JA, et al. Physical activity, quality of life, and weight status in overweight children. Qual Life Res. 2008;17(3):407–12. 10.1007/s11136-008-9312-y 18293100

